# Netrin 1 and Alpha-Methyl Acylcoenzim-A Racemase in diagnosis of prostate cancer

**DOI:** 10.25100/cm.v49i2.3011

**Published:** 2018-06-30

**Authors:** Ersin Koseoglu, Altug Tuncel, Melih Balci, Oguzhan Kaya, Yilmaz Aslan, Ozer Guzel, Fatma Meric Yilmaz, Ali Atan

**Affiliations:** 1 Ankara Numune Research and Training Hospital, Department of Urology. Ankara, Turkey; 2 Ankara Numune Research and Training Hospital, Department of Biochemistry. Ankara,Turkey

**Keywords:** Netrin 1, alpha-methyl acylcoenzim-A racemase, prostate, cancer, Netrina 1, alfa-metil acilcoenzima-A racemosa, cáncer, próstata

## Abstract

**Objectives:**

To investigate serum and urine levels of Alpha-methylacyl-CoA-racemase (AMACR) and Netrin 1 in patients with and without prostate cancer and to determine whether these markers could be used as alternatives in diagnosis of prostate cancer instead of serum prostate specific antigen (PSA) levels.

**Methods:**

One hundred and seventy five patients between 45-75 years to whom transrectal ultrasound guided biopsies were performed for abnormal serum PSA levels or digital rectal examinations were included. The levels of AMACR and Netrin 1 levels of blood and urine samples of 5 mL those were taken prior to biopsies were measured. .

**Results:**

The mean age of the patients was 62.7 ±6.4 years. Prostate cancer was detected in 40 patients (22.8%) while 135 of them (77.2%) were diagnosed as benign prostate hyperplasia (BPH). In BPH group, serum and urine levels of AMACR and Netrin 1 were 13.4 ±16.9 ng/mL; 7.1 ±3.4 ng/mL; 164.1±46 pg/mL and 19.5 ±5.0 pg/mL respectively. The levels of serum and urine levels of AMACR and Netrin 1 were 10.2 ±9.8 ng/mL; 6.8 ±2.5 ng/mL; 159.1 ±44.1 pg/mL and 20.1 ±5.3 pg/mL respectively in prostate cancer group. There was no statistically significant difference or correlation between these two groups serum and urine AMACR and Netrin 1 results

**Conclusions:**

Serum and urine levels of AMACR and Netrin 1 were not found to be alternatives for serum PSA levels in the diagnosis of prostate cancer in this study.

## Resumen

**Objetivos:**

Investigar los niveles de alfa-metil acilcoenzima-A y Netrina 1 en pacientes con y sin cáncer de próstata y determinar si estos marcadores pueden ser usados como una alternativa en el diagnóstico de cáncer de próstata en lugar del antígeno prostático específico en suero (PSA).

**Métodos:**

Fueron incluidos 175 pacientes entre 45-75 años, a quienes se les realizó una biopsia de próstata guiada por ultrasonido por presentar un nivel anormal de PSA en el suero o un tacto rectal. Se tomó una muestra de 5 mL de sangre y orina para medir los niveles de alfa-metil acilcoenzima-A y Netrina 1. Estos niveles se midieron antes del análisis de la biopsia.

**Resultados:**

La edad media de los pacientes fue de 62.7±6.4 años. Se detectó cander en 40 pacientes (22.8%), mientras que a 135 de ellos (77.2%) se les diagnóstico una hiperplasia benigna de próstata (HBP). En el grupo HBP los niveles en suero y orina de alfa-metil acilcoenzima-A y Netrina 1 fueron 13.4 ±16.9 ng/mL; 7.1 ±3.4 ng/mL; 164.1 ±46 pg/mL y 19.5 ±5.0 pg/mL respectivamente. En el grupo con cáncer de próstata los niveles en suero y orina de alfa-metil acilcoenzima-A y Netrina 1 fueron 10.2 ±9.8 ng/mL; 6.8 ±2.5 ng/mL; 159.1 ±44.1 pg/mL y 20.1 ±5.3 pg/mL respectivamente. No hubo una diferencia significativa o una correlación entre los niveles de alfa-metil acilcoenzima-A y Netrina 1 en suero y orina al comparar estos dos grupos de pacientes.

**Conclusiones:**

Los niveles de alfa-metil acilcoenzima-A y Netrina 1 en suero y orina no son una alternativa para reemplazar el PSA en suero para el diagnóstico de cáncer de próstata.

## Introduction

Prostate cancer (PCa) is the most common malignancy among elderly (>70 years) males in Europe. This disease is particularly a major health problem in industrialized countries whose majority of population is composed of elderly population ^1^. Nowadays, prostate specific antigen (PSA) and digital rectal examination (DRE) are the initial steps on the way of screening and diagnosis for the PCa. For the patients with abnormal results and findings on these steps, generally transrectal ultrasonography (TRUSG) guided prostate biopsy is performed for pathological diagnosis. However, the low specificity rate of PSA leads to high false negative prostate biopsy results ^2^. Also, slow progressing or aggressive PCa could not be differentiated with PSA, and this may lead to overtreatment and misuse of healthcare resources ^3^. Therefore, countless efforts have been made to developed a new biomarker that would improve current diagnostic methods and identify patients with lethal PCa.

Alpha-methylacyl-CoA-racemase (AMACR) is an enzyme known to play a key role in the peroxysomal beta oxidation of branched fatty acid chain ^4^. Although AMACR is not prostate specific, tissue sampling studies have shown that its levels are found to be elevated much more in PCa than in benign prostatic diseases ^5^ ^-^ ^7^.

Netrin 1 which resembles the extracellular matrix protein laminin, guide the axonal conduction. This marker has been reported to be secreted outside the nervous system, play a role in the epithelial tissue development, adhesion, motility and proliferation. Netrin 1 affects cellular differentiation, inflammation, angiogenesis and regulates apoptosis. Secretion of Netrin 1 increases during malignant processes due to cancer regression ^8^.

In this study we evaluated the levels of serum and urine PSA, AMACR and Netrin 1. The aim was to determine whether AMACR and Netrin 1 could be used instead of serum PSA in diagnosis of PCa.

## Materials and Methods

After the ethical committee approval, 175 patients between the ages of 45-75 years; serum PSA levels ≥2.5 ng/mL and/or abnormal DRE findings were planned to be prospectively included in our study. Informed consents were obtained from all patients. Serum PSA levels and urine analysis were planned to be studied on the first visit. Inclusion criteria were males with serum total PSA level above 2.5 ng/mL without infectious findings in the urine analysis. PCa diagnosis, presence of previous prostate biopsies, bleeding disorders, previous prostatic surgeries, uncontrolled urinary tract infections, being at age above 75 years were the exclusion criteria for our study. All patients were given Ciprofloxacin 1,000 mg/day p.o. 48 hours before TRUSG guided prostate biopsy for infection prophylaxis and continued 5 days after the procedure.

Before the biopsy procedure a 5 mL of serum sample and a urine sample after prostatic massage were obtained from the patients. In these samples AMACR and Netrin 1 levels were studied in the same laboratory. The urine and serum samples were collected in 5 mL vacuum blood tubes (BD Vacutainer, Clot Activator Tube, REF 368815, PL6 78P, Plymouth, England). The biochemistry tubes were centrifuged for 10 min with 4,000 rpm in Bench Top Centrifuge NF™ 1200R 02-05063000 centrifuge machine (Nuve Sanayi Hizmetleri, Ankara, Türkiye). The samples obtained were transferred into polypropilene eppendorf tubes and stored in ultra-low temperature freezer (Ultra-Low Temperature Freezer, MDF- U5386S, SANYO Electric Co., Ltd., Osaka, Japan) at -80° C. After the completion of the required number of patients all samples were removed from the freezer and left on room temperature to be ready for analysis. All of the tubes were given separate numbers and analyzed in Ankara Numune Training and Research Hospital, Biochemistry Laboratory. Serum and urine Netrin 1 levels were measured with Human Ntn1™ kit (Elabscience Biotechnology Co, Beijing, Peoples Republic of China); serum and urine AMACR levels were measured with Human AMACR ELISA™ kit (Hangzhou Eastbiopharm Co, Hangzhou, Peoples Republic Of China). For the measurement, Sandwich-ELISA method was performed with the use of washer (BioTek Instruments, Inc. Highland Park, Chicago, USA) and reader (BioTek Instruments, Inc. Highland Park, Chicago, USA). All samples and standards were transferred to the wells with a biotin-conjugated polyclonal antibody specific to AMACR and Netrin 1. Spectrophotometric measurements were performed by microplate reader set to 450 nm. Serial dilutions were prepared from a 100 ng/mL stock solution.

Prostate volume was measured in all of 175 patients using TRUSG. The prostate ellipsoid formula (*π/6 x longitudinal diameter x transvers diameter x anteroposterior diameter*) was used for the measurement of the prostate volume. Subsequently, 10 core prostate biopsies were obtained from each patient from the right apex, right middle, right basal, right lateral, right farlateral, left apex, left middle, left basal, left lateral, left farlateral parts. Serum and urine samples of the patients diagnosed with PCa after pathological evaluation and those without PCa were evaluated.

### Statistical analysis

Statistical Package for Social Sciences for Windows (SPSS®, Chicago, USA) 13.0 version was used for statistical analysis. Descriptive statistics were obtained and demographical features were given as mean ± standard deviation. Data with normal distribution (age, serum AMACR and serum Netrin 1) were compared with *Student t test*, and data with abnormal distribution (serum PSA, total prostatic volume, urine AMACR and urine Netrin 1) were compared with *Mann Whitney-U test.* Pearson correlation analysis was performed for the correlation between serum and urine AMACR and Netrin 1 levels, and between serum PSA levels and the Gleason score. *p* <0.05 was considered statistically significant.

## Results

The mean age of the patients was 62.7 ±6.4 (45-75) years. According to the histopathological evaluation of the TRUS guided prostatic biopsy, 40 patients (22.8%) had PCa and 135 (77.2%) patients had with benign prostatic hyperplasia (BPH). The mean age of the BPH patients was 62 ±6.1 (48-75) years while the patients with PCa was 63.6 ±7.1 (51-74) years. No significant age difference was found between the two groups (*p*= 0.533).

Average serum PSA levels in BPH and PCa groups were 9.2 ng/mL and 17.6 ng/mL respectively (*p*= 0.041). According to the histopathological evaluation, from the patients with PCa, 31 (77.5%) had Gleason score 6.5 (12.5%) had Gleason score 7.1 (2.5%) had Gleason score 9, and 2 (5.0%) patients had Gleason score 10.

The mean prostate volumes measured with TRUS of the patients with BPH and PCa were 57 ±29.0 cm^3^ (17-193 cm^3^) and 47. 9±18.6 cm^3^ (17-91 cm^3^) (*p*= 0.072) respectively. 

In the BPH group average serum AMACR, urine AMACR, serum Netrin 1 and urine Netrin 1 levels were 13.4 ±16.9 ng/mL (2.8-92.4 ng/mL); 7.1 ±3.4 ng/mL (1.5-36 ng/mL); 164.1 ±46.0 pg/mL (29.6-281.7 pg/mL) and 19.5 ±5.3 pg/mL (11.8-37.0 pg/mL) respectively. In the PCa group, average serum AMACR, urine AMACR, serum Netrin 1 and urine Netrin 1 levels were 10.2±9.8 ng/mL (21.9-49.5 ng/mL); 6.8 ±2.5 ng/mL (3.2-14.9 ng/mL); 159.1 ±44.1 pg/mL (38.1-225.9 pg/mL) and 20.1 ±5.3 pg/mL (11.5-33.6 pg/mL) respectively (Table 1).

When serum and urine AMACR levels were compared in both groups, although they were lower in patients with PCa, this difference was statistically insignificant (*p*= 0.254 and *p*= 0.676) (Table 1).

Serum Netrin 1 levels were found to be higher in BPH group compared to PCa group, however, this difference was statistically insignificant (*p*= 0.542). Urine Netrin 1 levels in PCa group compared to the other group were higher however this difference was statistically insignificant (*p*= 0.656) (Table 1).

**Table 1:** The mean levels of serum AMACR, urine AMACR, serum Netrin 1 and Urine Netrin 1 levels in BPH and PCa groups with p values when compared.

	Serum AMACR (ng/mL)	Urine AMACR (ng/mL)	Serum Netrin 1 (pg/mL)	Urine Netrin 1 (pg/mL)
BPH	13.4±16.9	7.1±3.4	164.1±46.0	19.5±5.3
PCa	10.2±9.8	6.8±2.5	159.1±44.1	20.1±5.3
*p*	0.254	0.676	0.542	0.656

In the correlation analysis no correlation was found between serum AMACR and Netrin 1, urine AMACR and Netrin 1 levels and serum PSA levels of PCa patients (r_serum AMACR_= 0.019; r_serum Netrin 1_= -0.155; r_urine AMACR_= 0.060; r _urine Netrin1_= 0.646) (Table 2). No correlation has been found between serum AMACR, serum and urine Netrin 1 levels and Gleason scores (r_serum AMACR_= -0.159; r_serum Netrin 1_= 0.063; r_serum Netrin 1=_ -0.100). However, positive correlation was detected between urine AMACR levels and the Gleason score (Gleason <7) (r_urine AMACR_= 0.344) *(* Table 3).

**Table 2:** The correlation of serum AMACR, urine AMACR, serum Netrin 1, urine Netrin 1 and serum PSA levels fort he patients with prostate cancer

Parameters	PSA	
Serum AMACR	r	0.019
	p	0.909
Urine AMACR	r	-0.155
	p	0.341
Serum Netrin 1	r	0.060
	p	0.714
Urine Netrin 1	r	0.046
	p	0.776

r: Pearson correlation coefficient

**Table 3:** The correlation of serum AMACR, urine AMACR, serum Netrin 1, urine Netrin 1 and Gleason scores for the patients with prostate cancer

Parameters	Gleason Score	
Serum AMACR	r	-0.159
	p	0.328
Urine AMACR	r	0.344*
	p	0.030*
Serum Netrin 1	r	0.063
	p	0.698
Urine Netrin 1	r	-0.100
	p	0.539

*r: Pearson correlation coefficient*

* Significant

In the analysis performed without considering the histopathological diagnoses; patients with serum PSA levels above 10 ng/mL (n= 53; n_PCa_= 21; n_BPH_= 32) and below 10 ng/mL (n= 122; n_PCa_= 14; n_BPH_= 108) had no difference in serum AMACR, serum Netrin 1, urine AMACR and urine Netrine 1 levels (p_serum AMACR_= 0.781; p_serum Netrin 1_= 0.188; p_urine AMACR_= 0.339; p_urine Netrin1_= 0.343). Of the 40 patients with PCa; no correlation was found between urine AMACR and Netrin 1 levels and serum PSA ≥10 ng/mL and < 10 ng/mL levels (p_serum AMACR_=0.735; p_urine AMACR 1_=0.379; p_serum Netrin_ =0.250; p_urine Netrin1_= 0.786).

## Discussion

 Prostate-Specific Antigen is the most frequently used diagnostic laboratory tool for PCa ^9^. The advantages of PSA are: its specificity for prostate, good prediction value in PCa metastases, cost effectivity and simple clinical usage. However, its disadvantages are; not being specific to PCa, low sensitivity in early stage cancer detection, many factors other than PCa may affect its serum levels and it is insufficient in the differentiation of clinically insignificant versus agressive PCa. Therefore there are ongoing studies of new serum and urine markers that would substitute PSA in the diagnosis and follow-up of PCa ^10^.

 Alpha-methylacyl-CoA-racemase is an enzyme that transforms branched chain fatty acids and bile acid through beta oxidation to dihydroxy cholestanol and trihydroxy cholestanol thus transforming α-methyl branched chain fatty acids to CoA and S stereoisomers, and it is produced in the prostate in large amounts^5^. After it was concluded that AMACR enzyme is overexpressed in PCa, some studies have been conducted regarding AMACR expression in PCa diagnosis ^5^ ^,^ ^11^ ^,^ ^12^. Jiang *et al*. ^13^, assessed AMACR as a molecular biomarker for prostate cancer. The authors used a monoclonal antibody to stain 137 prostatic cancer tissue samples and 70 benign prostate tissue samples. They reported positive expression of AMACR in all 137 prostate cancer specimens, and analysis revealed a sensitivity of 100 % and specificity of 88%. A study by Rubin *et al*. ^14^, similarly demonstrated overexpression of AMACR in prostate cancer, evaluation of AMACR protein expression in 94 prostate needle biopsy specimens demonstrated 97% sensitivity and 100% specificity for detecting prostate cancer. However, there are no sufficient international studies regarding serum and urine AMACR levels in PCa diagnosis. Sreekumar et al. have stated that detecting serum AMACR is very difficult because of its low serum levels. They have measured serum AMACR levels through AMACR autoantibodies via ELISA technique in PCa. The immune response to AMACR of 54 patients with PCa was found to be higher compared to the control group of 55 participants. No correlation was detected between serum AMACR autoantibody and PSA levels, Gleason score and biochemical recurrence ^15^. In this study patients with additional oncological or immunological disorders were not excluded from the study, therefore it is not known whether AMACR autoantibody levels have been affected. In our study however, 40 patients diagnosed with PCa, but without other known oncological or immunological diseases, had lower serum AMACR levels compared to the serum AMACR levels of BPH patients. However, this difference was statistically insignificant (*p*= 0.254). In Sroka et al’s study, AMACR levels of 33 PCa patients and 38 BPH patients analyzed in urine samples before and after digital prostate massage were found to be higher in the PCa group. It has been reported that there was no correlation between the tumor stage and Gleason score. As a result, urine AMACR levels had no superiority over PSA in PCa diagnosis ^12^. In our study, urine AMACR levels of PCa patients were found to be lower when compared to BPH patients, without any statistical significance (*p*= 0.254). Unlike Sroka *et al*., study a low positive correlation was found between high Gleason score (≥7) and urine AMACR levels (*p*= 0.030; r= 0.344). However, in our study the patients with Gleason score <7 constituted 77.5% (n= 31/40) of all patients with PCa, therefore these results should be verified with further studies.

It is known that Netrin 1 expression increases in breast cancer, colorectal, lung, melanoma, pancreas and brain cancer (glioblastoma) ^16^ ^-^ ^20^. Also it has been shown that decrease in Netrin 1 receptor expression plays an important role in the development and progression of cancer (in angiogenesis, neurogenesis, tissue morphogenesis, embryonic development, cancer, inflammation, and various pathologies) in different solid organs ^8^ ^,^ ^21^. Kong *et al*. ^21^, found that netrin-1 was highly expressed in the nucleolus of prostate cancer cell lines and the authors postulated that higher netrin-1 and lower UNC5B expression in all prostate carcinoma cell lines indicated that netrin-1 and UNC5B could be used to predict metastasis. Chen *et al*. ^21^, indicate that netrin-1 may function as a positive regulator of hypoxia-triggered malignant behavior in PCa by activating the Yes-associated protein signaling. Accordingly, netrin-1 could be a promising therapeutic agent against prostate carcinoma. However, it is not clear if this increase in expression makes changes in serum and urine Netrin 1 levels. In the sole study regarding serum Netrin 1 levels in PCa diagnosis ^8^, serum Netrin 1 levels of 40 patients with PCa were evaluated with ELISA and compared with a control group of healthy participants. In the present study, average serum Netrin 1 levels were found to be significantly higher. However, in the previously mentioned study the PSA levels of the control group were not reported and correlation analysis was not performed between the Gleason scores and serum Netrin 1 levels of the PCa patients. In our study unlike the mentioned average serum Netrin 1 levels of the PCa group were found to be lower than the BPH group. However, this difference was statistically insignificant. Furthermore, no correlation was detected between 9 patients with Gleason score ≥7 and 31 patients with Gleason score <7 regarding Netrin 1 levels.

There is no study in the literature to our knowledge evaluating Netrin 1 levels in PCa patients. Our study is the first in the literature comparing urine Netrin 1 levels in PCa and BPH patients. In our study, PCa patients’ mean Netrin 1 levels were lower than BPH patients. However, these differences were statistically insignificant (*p*= 0.656). No significant correlation was found between Gleason score and Netrin 1 levels in our study.

No study in the literature to our knowledge has evaluated the correlation between serum PSA levels and urine and serum AMACR and Netrin 1 levels. In the present study, no significant correlation was found between serum and urine AMACR and Netrin 1 levels in patients with serum PSA <10 ng/mL and ≥10 ng/mL. Furthermore, there was no significant correlation between PSA levels and serum and urine AMACR and Netrin 1 levels in PCa patients.

Our study has the feature of being the “first” evaluating Netrin 1 levels in urine in PCa. Furthermore, it is among the few studies evaluating serum AMACR and Netrin 1 and urine AMACR levels in PCa. The disadvantages of this study are; the small number of PCa patients, being single centered study and the lack of comparison of tissue expression and results of the markers that were evaluated.

There is no data in the literature to our knowledge regarding the relation between AMACR and Netrin 1 levels, and age and prostate volume. We think that the lack of difference between the average age of PCa and BPH groups is of importance as it does not support the age related hypothesis. Also, the similar prostate volumes measured by TRUSG may be an important data showing that the levels of these markers are not affected by the prostate volume.

## Conclusion

It was shown that serum and urine AMACR and Netrin 1 levels could not replace PSA measurement as an alternative for PCa diagnosis. Larger study groups, multicentered studies and tissue expression analysis of AMACR and Netrin 1 could give a lead on new hopes in PCa diagnosis.
